# A Rapid, Accurate, Single Molecule Counting Method Detects *Clostridium difficile* Toxin B in Stool Samples

**DOI:** 10.1038/s41598-018-26353-0

**Published:** 2018-05-30

**Authors:** Sadanand Gite, Destiny Archambault, Michael P. Cappillino, David Cunha, Victoria Dorich, Tatyana Shatova, Andrew Tempesta, Bruce Walsh, Jessica A. Walsh, Adam Williams, James E. Kirby, Jayson Bowers, Don Straus

**Affiliations:** 1 First Light Biosciences, 1 Oak Park Drive, Bedford, MA 01730 USA; 2 Beth Israel Deaconess Medical Center, 330 Brookline Ave, Boston, MA 02215 USA

## Abstract

We describe a new rapid and accurate immunoassay-based technology capable of counting single target molecules using digital imaging without magnification. Using the technology, we developed a rapid test for *Clostridium difficile* toxin B, which is responsible for the pathology underlying potentially fatal *C. difficile* infections (CDI). There are currently no tests for CDI that are rapid, sensitive, and specific. The MultiPath *C. difficile* toxin B test images and counts complexes of target-specific magnetic and fluorescent particles that have been tethered together by toxin B molecules in minimally processed stool samples. The performance characteristics of the 30 minute test include a limit of detection of 45 pg/mL, dynamic range covering 4–5 orders of magnitude, and coefficient of variation of less than 10%. The MultiPath test detected all toxinotypes and ribotypes tested, including the one most commonly occurring in the US and EU; shows no cross reactivity with relevant bacterial species; and is robust to potential interferants commonly present in stool samples. On a training set of 320 clinical stool samples, the MultiPath *C. difficile* toxin B test showed 97.0% sensitivity (95% CI, 91.4–99.4%); 98.3% specificity (95% CI, 96.8–99.2%); and 98.2% accuracy (95% CI, 96.7–99.0%) compared to the cellular cytotoxicity neutralization assay (CCNA) reference method. Based on these compelling performance characteristics, we believe the MultiPath technology can address the lack of rapid, sensitive, specific, and easy-to-use diagnostic tests for *C. difficile*.

## Introduction

The ability to detect and quantify biologically important proteins is essential for medical diagnostics. In the case of infectious disease diagnostics, detection of pathogen-specific proteins is a commonly used method to identify diseases. Examples include tests for influenza, hepatitis B, human immunodeficiency virus, *Legionella*, *Cryptococcus*, *Plasmodium* antigens, and the secreted toxins associated with *C. difficile* colitis. Technologies currently used in clinical laboratories include automated enzyme-linked immunosorbent assays (ELISAs) and rapid lateral flow tests. However, current immunoassay technology has several limitations with respect to assay sensitivity, speed, ability to multiplex, and/or ease of use. The low sensitivity of standard immunoassay technology has limited its use and utility in the detection of infectious diseases where antigen concentrations are low. An example of this is the insufficient sensitivity (50–70%) of the influenza antigen detection test widely used in point of care settings^1^. Similarly, current insensitive *C. difficile* toxin assays have precluded reliable use in diagnosis of *C. difficile* colitis and have led to current clinical reliance on alternative technologies which have less than desirable performance characteristics. *C. difficile* colitis afflicts approximately 453,000 patients and results in approximately 29,000 deaths annually in the USA^2^. It is caused by action of a cytotoxin elaborated by the anaerobic *C. difficile* on the large intestinal epithelium. Infections can be life-altering, recurrent, and often require repeated courses of therapy and even stool transplant for cure. Approximately 2% of the normal population are colonized by *C. difficile*. However, it is the toxin, not the mere presence of the organism, that is associated with disease.

As such, patients with active *C. difficile* infection can be distinguished from colonization by testing for toxin production. However, current commercially available toxin testing platforms such as enzyme immunoassays (EIAs) are insensitive^3,4^. An alternative test, the tissue culture based cytotoxicity assay is sensitive, but takes 1–3 days to perform and is highly complex. It is therefore largely used only as a reference method. Other tests in clinical practice include nucleic acid amplification tests (NAATs). Although, NAATs are highly sensitive, they are also non-specific, as they detect *C. difficile* toxin genes regardless of toxin production. Their use leads to clinical confusion, overtreatment, and potential delays in diagnosis of alternative illnesses^5^. Some algorithms combine a sensitive test, such as a NAAT or an antigen test for the *C. difficile* enzyme glutamate dehydrogenase, with a more specific antigen test for toxins^6^. However, these algorithms do not fully address the shortcomings of the individual test components and create undesirable complexities for diagnostic laboratories.

Therefore, an ideal diagnostic test for *C. difficile* colitis would be a rapid and highly sensitive toxin antigen detection test, as it should prove to be both sensitive and specific for *C. difficile*-associated disease^3,7^. This notion has been supported by the recent update to clinical practice guidelines for diagnosing *C. difficile* infections^8^. Several such rapid and sensitive immunoassay technologies are being developed but are not yet in commercial use^7,9^. Examples include immunoassay technologies that detect modulation of total internal reflection, single molecules in femtoliter wells, and single molecules flowing through capillary tubes^10–12^. Furthermore, new technologies based on electrochemical, nanomechanical, or piezoelectric detection, or supersensitive optical detection may have potential application in next-generation immunoassay development^13,14^. Clinical translation and broad adoption of these emerging technologies depends on their ability to deliver optimal performance characteristics in a rapid, easy-to-use, and affordable platform.

Here, we present data demonstrating the potential of the novel MultiPath immunoassay technology to deliver a highly sensitive test for *C. difficile* toxin B antigen. The technology enumerates single target molecules with simple optical equipment, minimal sample preparation, and rapid turnaround time in a sample-to-answer format. We demonstrate equivalence in performance to the highly sensitive toxin B cytotoxicity assay reference method used for regulatory clearance of *C. difficile* toxin tests.

## Results

### MultiPath technology

**Figure 1:**
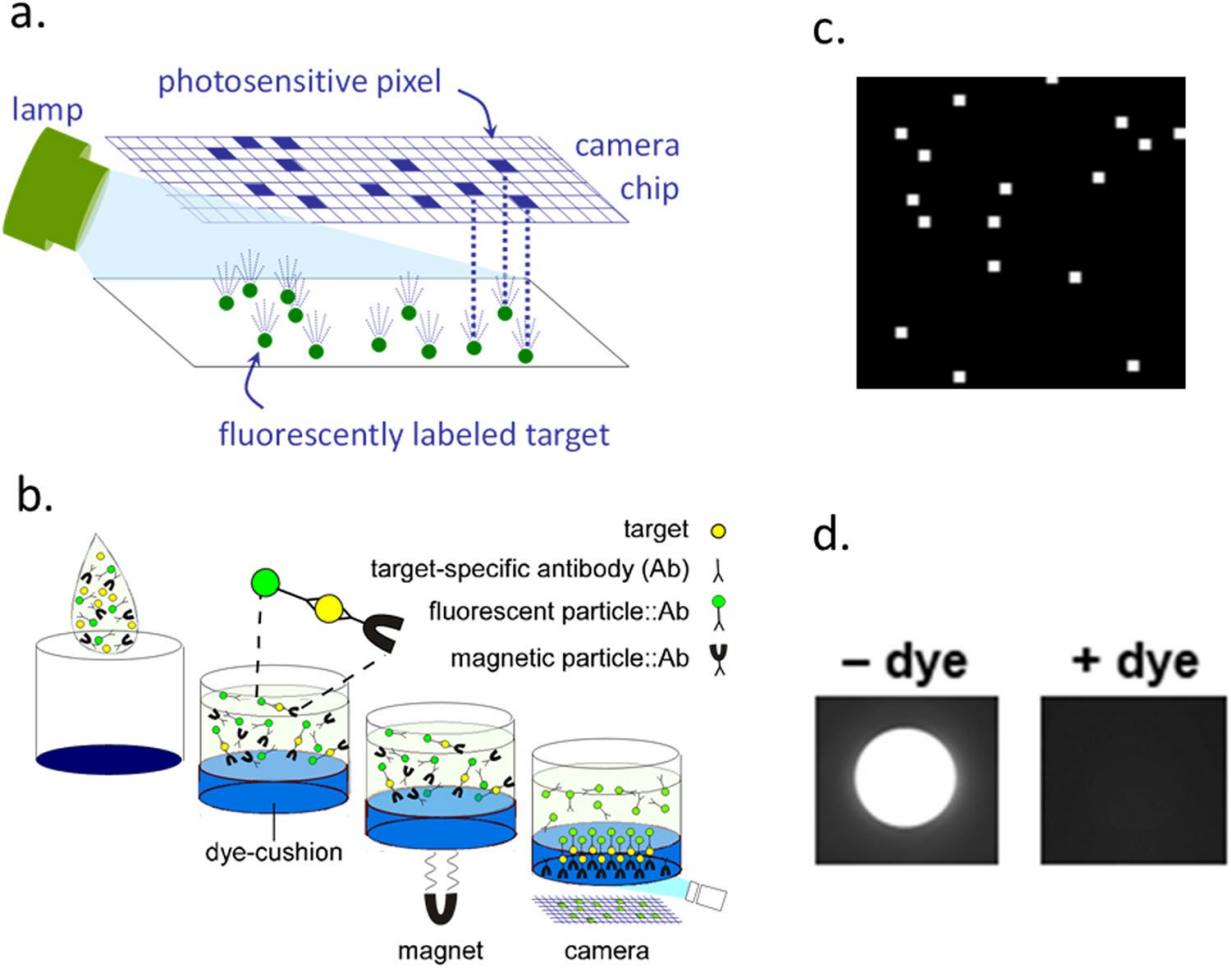
(**a**) The MultiPath technology uses non-magnified digital imaging to enumerate microscopic fluorescent particles bound to molecular targets. Light from the microscopic particles impinge on one or a small group of pixels on the camera’s chip creating a white spot in the image. (**b**) A non-magnified image from the *C. difficile* toxin B test showing individual microscopic fluorescent particles that have been tethered by toxin B molecules to magnetic particles, drawn through the dye cushion, and deposited on the imaging surface. (**c**) Target molecules tether target-specific fluorescent and magnetic particles together. Magnetic particles and any fluorescent particles bound to magnetic particles are drawn through the dense opaque dye-cushion layer and imaged. The dye-cushion in the assay’s imaging well eliminates the need for wash steps and minimizes sample preparation. (**d**) Images of wells containing unbound particles overlying cushion layers with or without dye demonstrate the effectiveness of the dye-cushion for eliminating background from the unbound fluorescent particles.

The MultiPath imaging technology detects molecules labeled with fluorescently dyed nanoparticles using digital imaging without magnification (Fig. 1a). Illuminating the fluorescent nanoparticle labels causes them to emit photons which are collected using a 1:1 f/4 relay lens. The light emitted by a particle impinges on a small cluster of pixels on the CMOS chip of a digital camera forming white spots in the resulting image (Fig. 1b). At low analyte concentrations, digitally counting individually labeled targets generates better signal to noise ratios compared to simply integrating the signal from the entire detection area. Non-magnified imaging allows a large field to be imaged, enabling detection of a small number of target molecules in a large volume of sample in milliseconds.

Figure 1c illustrates how the MultiPath technology can count specific targets in samples without requiring sample preparation or wash steps. Samples are first mixed with a diluent and target-specific immunoreagents, which consists of fluorescent and magnetic particles coated with complementary antibodies specific for the target (*C. difficile* toxin B in this work). The assay mixture is then added to a clear-bottomed microtiter well, the bottom of which has been coated with a dried dye-cushion reagent. The dye-cushion reagent is a mixture of a dye that absorbs visible light (Direct Black 19 in these experiments) and a density agent, iodixanol (OptiPrep™). The dried dye-cushion reconstitutes following addition of the assay mixture and forms a dense opaque aqueous layer underneath the assay layer. Light cannot penetrate the dye-cushion layer to the assay layer (Fig. 1d). This feature optically sequesters unbound fluorescent labels and sample matrix from the imaging surface. Therefore, the dye-cushion obviates the need for laborious sample preparation and wash steps required in other immunoassay formats to eliminate the background signal caused by unbound label and sample matrix components.

### Estimating the analytical performance of the MultiPath *C. difficile* toxin B test

**Figure 2:**
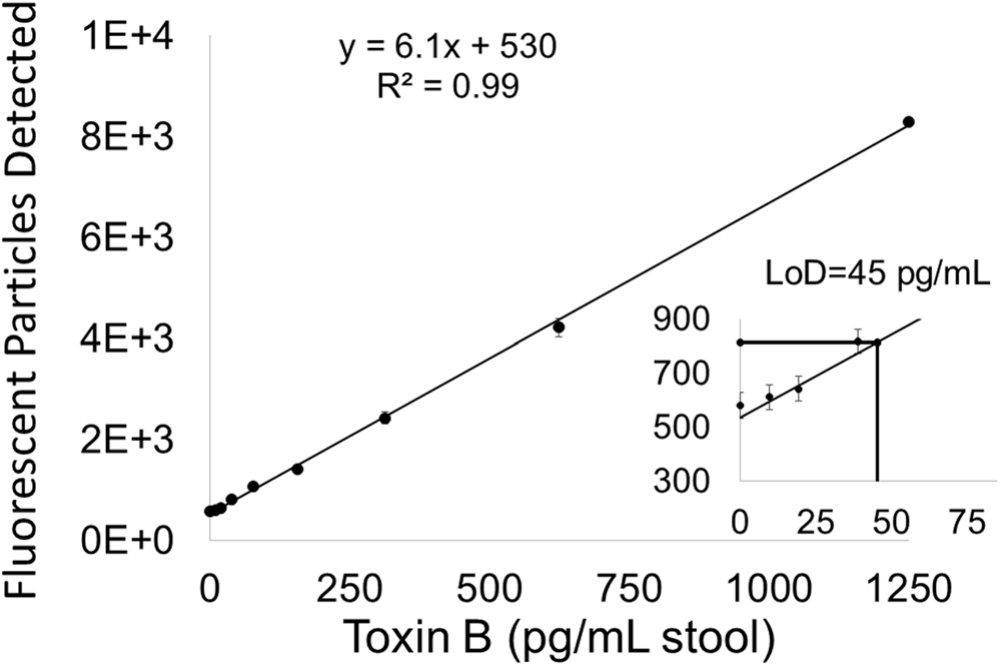
Analytical sensitivity of the *C. difficile* toxin B test. The y-axis reflects the signal. The x-axis reflects quantity of purified toxin B spiked into pooled stool samples. Error bars correspond to the standard deviations of the unspiked (n = 24) and spiked (n = 12) data points. The limit of detection is indicated in the inset.

**Figure 3:**
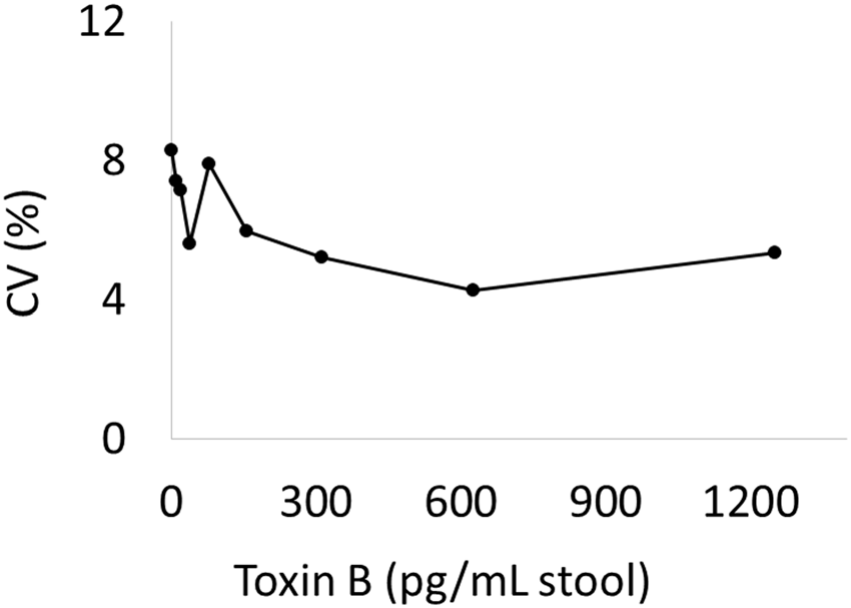
The precision profile of the *C. difficile* toxin B assay. The CV’s of the measurements from Fig. 2 are shown.

To develop a MultiPath assay specific for *C. difficile*, we conjugated magnetic and fluorescent particles to complementary murine monoclonal antibodies raised against *C. difficile* toxin B. When a stool sample containing toxin B is combined with these reagents, the toxin B molecules will bind to and tether the magnetic and fluorescent particles, which then can be counted as described above (Fig. 1). To estimate the analytical sensitivity of the MultiPath *C. difficile* toxin B test in stool matrix, we used a pooled stool sample comprised of 14 randomly chosen clinical samples that gave negative results when tested by a real-time PCR *C. difficile* test. We tested the pooled sample spiked with *C. difficile* toxin B in a series of two-fold dilutions. Figure 2 shows that the method delivered a limit of detection of 45 pg/mL for *C. difficile* toxin B. Similar results were also observed when we used a different pool of PCR negative stool samples (data not shown). At a toxin B concentration of 45 pg/mL, the reaction contains approximately 100-fold excess of magnetic particles compared to the number of toxin B molecules. At this analyte concentration, magnetic and fluorescent particles must, on average, be tethered together by single toxin B molecules confirming that the MultiPath technology detects single molecules by imaging without using magnification. Furthermore, the precision profile in Fig. 3 shows coefficients of variation (CVs) below 10% for the data shown in Fig. 2, demonstrating the method’s potential for delivering reproducible results at low concentrations of toxin B.

**Figure 4:**
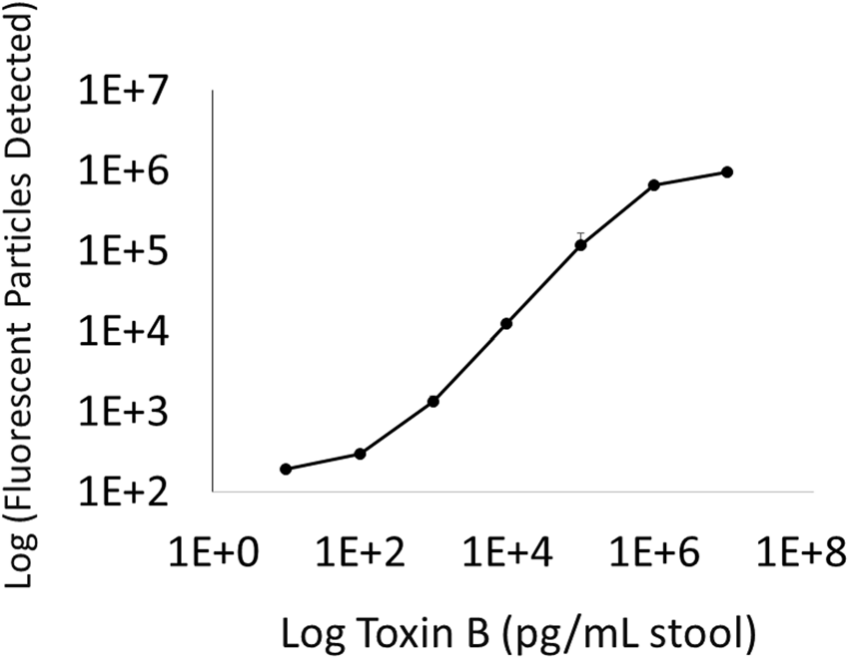
The dose response and dynamic range of the *C. difficile* toxin B assay. Purified toxin B protein was spiked into a mixture of pooled stool samples at the indicated concentrations. Error bars correspond to the standard deviations of 6 replicates.

Figure 4 shows the *C. difficile* toxin B test dose response in a pooled stool sample containing a range of concentrations of exogenously added purified *C. difficile* toxin B. The data are essentially linear over a concentration range of 4–5 orders of magnitude up to approximately 1 μg/mL, above which the response plateaus. Note that covering this range exceeds the highest levels of *C. difficile* toxin B reported clinically, about 100 ng/mL^15^.

### Assay controls detect and mitigate matrix effects

We designed positive and neutralizing assay controls to facilitate detection and subsequent mitigation of sample matrix effects. The assay controls and the toxin B test were performed in parallel using equal aliquots of a mixture containing clinical sample and assay reagents. The positive control includes a defined amount of spiked toxin (100 pg). A deviation of the positive control signal that is lower than the expected level indicates negative assay interference (assay inhibition). The neutralization control contains toxin B neutralizing antibodies that competitively bind to toxin B in the clinical sample, making it undetectable in the assay. In this way, the neutralization control distinguishes specific signal derived from toxin B in the sample from non-specific signal. Non-specific signal can result from analyte-independent deposition of either fluorescent particles or auto-fluorescent sample components on the imaging surface. In this work, we used a training set of clinical samples to empirically establish signal, neutralization, and interference thresholds for optimizing diagnostic accuracy relative to the cytotoxicity assay reference method.

**Figure 5:**
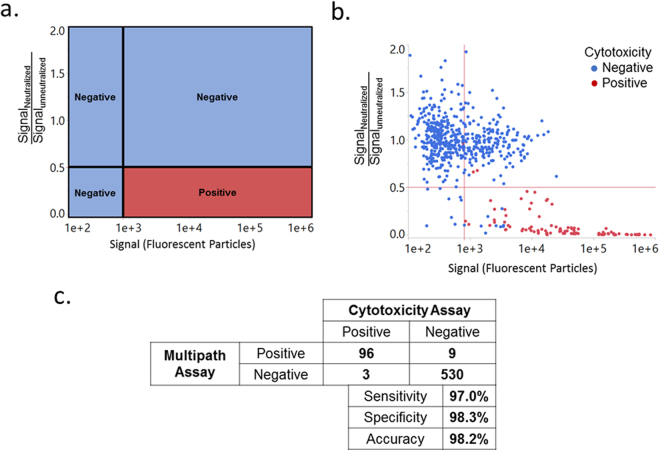
The results of the *C. difficile* toxin B test using clinical samples. The x-axis corresponds to the MultiPath signal for a clinical sample. The y-axis reflects the fraction of that signal remaining in the presence of neutralizing antibodies against toxin B. (**a**) is a graphical representation of the algorithm for calling positives and (**b**) shows the data from the study. Red dots represent samples that were called positive by the reference method (CCNA), and blue dots were negative by the reference method. (**c**) shows the sensitivity, specificity and accuracy of the MultiPath *C. difficile* toxin B test relative to the reference cytotoxicity assay.

Figure 5a graphically demonstrates the decision matrix for positive and negative calls by the *C. difficile* test. The decision matrix is comprised of 2 thresholds, a signal threshold (on the x-axis) and a neutralization threshold (on the y-axis). Only samples that exceed the signal threshold and that fall below the neutralization threshold are called positive. This is visualized as positive calls in the lower right-hand quadrant in Fig. 5a,b and negative calls for the other three quadrants. In addition, if interference is detected in the positive control (>75% change compared to the expected signal) the sample is declared invalid (data not shown).

### Accuracy of the MultiPath *C. difficile* toxin B test on clinical samples

Using the MultiPath *C. difficile* toxin B test described above, we analyzed 320 clinical stool samples from patients suspected of having *C. difficile* infection. Samples were tested in duplicate. The results from this sample training set were compared to the results of the toxin B cytotoxicity assay reference method. We used Receiver Operator Curve analysis to empirically develop assay thresholds for optimizing accuracy. Of the 320 clinical samples, only one sample (both replicates) was rejected from this analysis because it showed more than 98% inhibition of the positive control.

Figure 5b plots the training set results. The data illustrates that the chosen thresholds effectively distinguish positive and negative samples. Samples that scored positive using the cytotoxicity assay (red dots) fall almost entirely within the lower right-hand quadrant, representing samples with significant neutralizable signal. In contrast, samples that scored negative by the reference test (blue dots) fall almost entirely in one of the other three quadrants representing results that have either low signal, non-neutralizable signal, or both. Figure 5c compares the results of the MultiPath *C. difficile* toxin B test to the reference cytotoxicity assay. Using the chosen thresholds, the new method presented here achieved 97.0% sensitivity (95% CI, 91.4–99.4%); 98.3% specificity (95% CI, 96.8–99.2%); and 98.2% accuracy (95% CI, 96.7–99.0%) when compared to the cytotoxicity assay reference method.

We also performed analytical inclusivity, exclusivity, and interference studies which demonstrated that the test is robust to these variables (see data in supplementary results).

## Discussion

The MultiPath technology offers a novel method for rapid and sensitive detection of diagnostic markers directly in complex patient samples. We demonstrated the MultiPath technology’s potential by designing a rapid and accurate test for the life-threatening gastrointestinal infection caused by *C. difficile*, which causes more health-care associated infections than any other microbial pathogen^16^. The analytical sensitivity of the 30 min *C. difficile* toxin B test in stool samples is 45 pg/ml, which is more than 15 times more sensitive than the leading EIAs^17,18^. We also showed that the accuracy of the clinical sample results is high when compared to CCNA, a sensitive reference method with an analytical sensitivity of down to about 1.5 pg/ml in the absence of sample matrix^19^. The unique dye cushion format allows detection with minimal sample processing because it optically sequesters the sample and unbound label from the imaging surface. Stool is simply diluted and passed through a nylon mesh filter to remove large particulates before adding the immunoreagents. The simple sample preparation and lack of wash steps offer the potential to eliminate significant hands-on time, lower costs, and simplify instrumentation when compared to other EIAs.

The study’s limitations include the fact that we only tested for *C. difficile* toxin B and not toxin A. While there is conflicting data on the relative roles of toxins A and B in *C. difficile* infection, current evidence implicates *C. difficile* toxin B as being responsible for the most severe symptoms of *C. difficile* infection^20,21^. Furthermore, apparently few, if any, toxin A+/B− strains have been isolated from patients with CDI, whereas toxin A−/B+ strains have been isolated from patients with CDI^22,23^. In this study, we only used unformed stool samples. Although published guidelines recommend that testing be limited to unformed stool samples^4^, in practice formed stool samples are sometimes tested. We did test a limited number of formed and semi-formed stool samples and found that our method yielded results comparable to the CCNA reference method (data not shown). The study was also limited by its use of de-identified patient samples. Thus, we could not perform sub-analysis based on patient information (*e.g*., age, sex, medical history, clinical data, laxative use, or antibiotic use). The study also did not use fresh samples, and therefore fresh samples will need to be examined in future studies to rule out potentially labile interferents in sample matrix. Finally, this study used clinical samples as a training set to establish thresholds that optimized the correlation with the CCNA reference method. Thus, the results from the training set may not be extensible to new samples. Since this was not a blinded study, it is potentially subject to bias. These latter limitations should be addressed by future blinded studies using pre-established thresholding parameters.

The MultiPath *C. difficile* toxin B test has the potential to address a gap in *C. difficile* infection diagnostics^3,4^. Since it directly detects the toxin that is only produced by growing vegetative *C. difficile* cells – and not the dormant spores which don’t cause disease – it can eliminate the false positives that lower specificity and positive predictive value of nucleic acid amplification tests. Because the new test’s Limit of Detection is significantly lower than on-market EIAs, it also has the potential to improve on the clinical sensitivity of tests based on that technology.

## Materials and Methods

### Reagents

Fluorescent microparticles (500 nm), 1-Ethyl-3-(3-dimethylaminopropyl) carbodiimide (EDC) and N-hydroxysuccinimide (NHS) were purchased from Thermo Fisher Scientific (Waltham, MA). Polystyrene carboxylate magnetic particles (292 nm) were purchased from Ademtech (Pessac, France). Magnets for capturing magnetic particles were from Dexter Magnetic Technologies (Elk Grove, IL). Microtiter plates (96 well clear bottom, half area black plate) were from Greiner Bio-One (Monroe, NC). Native Toxin B standard purified from *C. difficile* (ribotype 087) was purchased from List Laboratories (Campbell, CA). Mouse monoclonal antibodies raised against *C. difficile* toxin B were from BBI Solutions (Cardiff, UK) and Fitzgerald (Acton, MA). Bovine serum albumin (BSA), Casein, Trizma® base, Trizma®-HCl, Triton X-100 and OptiPrep were from Sigma-Aldrich (St. Louis, MO). Direct Black 19 was from Orient Corporation (Cranford, NJ). Protease inhibitor cocktail was from Takara Bio (Mountain View, CA).

### Clinical samples

De-identified discarded stool samples were obtained from Beth Israel Deaconess Medical Center (Boston, MA) and Discovery Life Sciences (Los Osos, CA). The samples were collected over a period of 12 months. We excluded formed stool samples (either semi-sold or solid) and samples from children under the age of 2. Samples were stored at 4 °C for 3–7 days in the clinical microbiology laboratory and then transferred to our laboratory in a cooler with ice or ice-packs to maintain > 4 °C. After receiving the samples, they were diluted to 40% with water and single use aliquots were made and stored at −80 °C until use. A pooled negative stool sample was made from 14 individual stool samples that had been scored as *C. difficile* negative by real-time PCR. Stool diluent (Tris buffer, casein, heterophilic blocking reagents and protease inhibitor cocktail) was added to the samples and the diluted stool samples were filtered through a 10-micron nylon mesh filter (PluriSelect, San Diego, US) prior to testing to remove particulates.

### Imaging system

The MultiPath laboratory imaging system is a custom-built instrument and software that is capable of automatically capturing image data from selected wells of a microtiter plate. It uses a high precision linear stage from Prior Scientific (Rockland, MA) to position each well over a fluorescence-based image acquisition subsystem. The instrument can image in 4 separate color channels and uses an objective lens, illumination LEDs, fluorescent filter sets, and camera. The objective lens has a field of view designed to capture the image of an entire microtiter plate well. The illumination module light source consists of 2 high power LEDs per color channel. A series of fluorescent image frames are captured with a camera using a 3.1MP Sony IMX265 monochrome sensor with 12-bit per pixel quantization. The final image for each well is then formed by summing multiple frames. For the *C. difficile* toxin B tests, we used 470/40 nm excitation and 515/30 nm emission filters and captured 2 frames at a 20 msec exposure.

### Preparation of microtiter plates containing dye cushion

Dye cushion was prepared by adding 50 µL of a solution containing 0.25 mg Direct Black 19, 10% (v/v) OptiPrep in 50 mM Tris-HCl, pH 7.5 to each well of a surface-plasma-treated 96-well microtiter plate and drying at 60 °C for 3 hours. Dried plates were stored desiccated for up to 1 month.

### Preparation of antibody-conjugated magnetic and fluorescent particles

Anti-toxin B monoclonal antibodies were conjugated to magnetic and fluorescent particles through a carboxyl linkage (EDC/NHS chemistry) using standard conjugation methods recommended by the particle manufacturers (Ademtech, PESSAC, France and Thermo Fisher Scientific, Waltham, MA). The conjugated magnetic particles were quantified by visible light absorption and the conjugated fluorescent particles were quantified using flow cytometry for the purposes of subsequent assay formulation.

### MultiPath *C. difficile* toxin B test

To prepare the assay mixture, stool was diluted to 8% with a mix of stool diluent, 7e8 particles/mL of antibody conjugated magnetic particles, 1.1e7 particles/mL of antibody conjugated fluorescent particles, and the indicated amount of toxin B (diluted in 50 mM Tris-HCl, pH 7.8 buffer containing 2 mg/mL BSA, 0.05% w/v Tween-20 and 0.05% v/v Proclin-300) or just buffer for blank. 100 µL of the assay mixture was pipetted in to each dried dye cushion-containing well. Following a 30 minute incubation at 35 °C, magnetic particles were pulled down by placing the assay plate on the Dexter magnet for 3 minutes. The plate was then imaged using a MultiPath imaging system, and the signal was quantitated as described below.

### Image analysis

The number of fluorescent particles were quantified for each acquired image using the MultiPath analysis algorithm as follows. The image was masked with a fixed pixel threshold creating a binary image where all pixels with intensities above the threshold are set to 1. Pixels from the image were grouped using connectivity analysis such that each active pixel was grouped with all active pixels that were immediately adjacent in either the x or y image direction. A pixel group, or blob, was then processed to determine a set of parameters such as area (number of pixels), blob intensity (total intensity of all pixels in a blob) and compactness $(\frac{perimete{r}^{2}}{4\pi area})$. The list of blobs was then filtered to remove non-specific signal which could be caused by the sample matrix. This was done by removing blobs based on size, intensity, and/or irregular shape. Once the blob list was filtered, the total blob intensity was computed by summing the intensity of each blob. The number of detected fluorescent particles was then computed by dividing the total blob intensity by the reference intensity of a single fluorescent particle.

### Limit of detection, limit of blank, dynamic range, and precision profile

These measurements were performed using the pooled negative stool sample. The limit of detection for the *C. difficile* MultiPath toxin B assay was determined by running 24 replicates of sample with no analyte and 12 replicates each of 7 toxin B concentrations. The limit of the blank, limit of detection, and precision profile were determined according to Clinical & Laboratory Standards Institute (CLSI) guidelines^24^.

### Testing *C. difficile* clinical samples

320 clinical samples were tested as described above using a 3 well assay (test, positive control, and neutralization control for each sample). Each sample examined was tested independently by two operators. For the positive control, assay mixture inclusive of patient sample was spiked with 100 pg/mL* C. difficile* toxin B to detect matrix inhibitory effects. For the neutralization control, the assay was spiked with 2.5 µg/mL *C. difficile* anti-toxin B antibodies to confirm that any signal in the un-neutralized sample was a result of toxin B detection.

### Cell Cytotoxicity neutralization assay (CCNA)

An aliquot of each sample that had been used for the MultiPath Test was sent frozen on dry ice to Microbiology Specialists Inc. (Houston, TX) for CCNA. On receiving, the integrity of the sample was checked and CCNA was performed using MRC-5 fibroblasts cells and Quidel cytotoxicity reagents (Quidel, Catalog number: 03-05000). Sample was diluted 5-fold using specimen diluent and centrifuged at 2000 to 6000xg for 10-minutes to pellet solid material. The supernatant was filtered through a sterile 0.45 micron membrane filter and filtrate was used for inoculating tissue culture plate with appropriate controls. The plate was incubated at 35 °C for 24–48 hours and the development of specific cytopathic effects was observed during the course of the incubation.

### Data analysis

Data were analyzed using JMP and GraphPad Prism software. Confidence intervals were determined using Clopper-Pearson analysis.

## Electronic supplementary material

Supplementary Material

## References

[1] Pollock NR (2009). Ruling out novel H1N1 influenza virus infection with direct fluorescent antigen testing. Clin Infect Dis.

[2] Lessa FC (2015). Burden of *Clostridium difficile* infection in the United States. N Engl J Med.

[3] Burnham CA, Dubberke ER, Kociolek LK, Polage CR, Riley TV (2016). *Clostridium difficile*-Diagnostic and Clinical Challenges. Clin Chem.

[4] Cohen SH (2010). Clinical practice guidelines for *Clostridium difficile* infection in adults: 2010 update by the society for healthcare epidemiology of America (SHEA) and the infectious diseases society of America (IDSA). Infect Control Hosp Epidemiol.

[5] Polage CR (2015). Overdiagnosis of *Clostridium difficile* Infection in the Molecular Test Era. JAMA Intern Med.

[6] Crobach MJ (2016). European Society of Clinical Microbiology and Infectious Diseases: update of the diagnostic guidance document for *Clostridium difficile* infection. Clin Microbiol Infect.

[7] Song L (2015). Development and Validation of Digital Enzyme-Linked Immunosorbent Assays for Ultrasensitive Detection and Quantification of *Clostridium difficile* Toxins in Stool. J Clin Microbiol.

[8] McDonald LC (2018). Clinical Practice Guidelines for *Clostridium difficile* Infection in Adults and Children: 2017 Update by the Infectious Diseases Society of America (IDSA) and Society for Healthcare Epidemiology of America (SHEA). Clin Infect Dis.

[9] Pollock NR (2016). Ultrasensitive Detection and Quantification of Toxins for Optimized Diagnosis of *Clostridium difficile* Infection. J Clin Microbiol.

[10] Jarrige V, Nieuwenhuis JH, van Son JP, Martens MF, Vissers JL (2011). A fast intraoperative PTH point-of-care assay on the Philips handheld magnotech system. Langenbecks Arch Surg.

[11] Todd J (2007). Ultrasensitive flow-based immunoassays using single-molecule counting. Clin Chem.

[12] Wilson DH (2016). The Simoa HD-1 Analyzer: A Novel Fully Automated Digital Immunoassay Analyzer with Single-Molecule Sensitivity and Multiplexing. J Lab Autom.

[13] Sang S (2016). Progress of new label-free techniques for biosensors: a review. Crit Rev Biotechnol.

[14] Dinarelli S, Girasole M, Kasas S, Longo G (2017). Nanotools and molecular techniques to rapidly identify and fight bacterial infections. J Microbiol Methods.

[15] Ryder AB (2010). Assessment of *Clostridium difficile* infections by quantitative detection of tcdB toxin by use of a real-time cell analysis system. J Clin Microbiol.

[16] Magill SS (2014). Multistate point-prevalence survey of health care-associated infections. N Engl J Med.

[17] Eastwood K, Else P, Charlett A, Wilcox M (2009). Comparison of nine commercially available *Clostridium difficile* toxin detection assays, a real-time PCR assay for *C. difficile* tcdB, and a glutamate dehydrogenase detection assay to cytotoxin testing and cytotoxigenic culture methods. J Clin Microbiol.

[18] Pollock NR (2015). Differential immunodetection of toxin B from highly virulent *Clostridium difficile* BI/NAP-1/027. J Clin Microbiol.

[19] Kelly CP (1996). Anti-*Clostridium difficile* bovine immunoglobulin concentrate inhibits cytotoxicity and enterotoxicity of *C. difficile* toxins. Antimicrob Agents Chemother.

[20] Carter GP (2015). Defining the Roles of TcdA and TcdB in Localized Gastrointestinal Disease, Systemic Organ Damage, and the Host Response during *Clostridium difficile* Infections. MBio.

[21] Tao L (2016). Frizzled proteins are colonic epithelial receptors for *C. difficile* toxin B. Nature.

[22] Kuehne SA (2010). The role of toxin A and toxin B in *Clostridium difficile i*nfection. Nature.

[23] Lyras D (2009). Toxin B is essential for virulence of *Clostridium difficile*. Nature.

[24] CLSI. *Evaluation of Detection Capability for Clinical Laboratory Measurement Procedures; Approved Guideline. CLSI document EP17-A2*. 2nd edn, (Clinical and Laboratory Standards Institute, 2012).

