# Supplementary material for: A Rapid, Accurate, Single Molecule Counting Method Detects Clostridium difficile Toxin B in Stool Samples
Source: Sci Rep. 2018 May 30;8:8364. doi: 10.1038/s41598-018-26353-0 (PMC5976643; doi:10.1038/s41598-018-26353-0)
Supplement: Supplementary file 1 — Supplementary Material [file 41598_2018_26353_MOESM1_ESM.docx]

A Rapid, Accurate, Single Molecule Counting Method Detects *Clostridium difficile* Toxin B in Stool Samples

Sadanand Gite^1,*^, Destiny Archambault^1^, Michael Cappillino^1^, David Cunha^1^, Victoria Dorich^1^, Tatyana Shatova^1^, Andrew Tempesta^1^, Bruce Walsh^1^, Jessica A. Walsh^1^, Adam Williams^1^, James E. Kirby^2^, Jayson Bowers^1^ and Don Straus^1^

^1^First Light Biosciences, 1 Oak Park Drive, Bedford, MA 01730 and ^2^Beth Israel Deaconess Medical Center, 330 Brookline Ave, Boston, MA 02215

# Supplementary Material

***Inclusivity studies****.* The *C. difficile* toxin B MultiPath assay was performed using 9 different toxin B samples purified from strains with diverse toxinotypes and ribotypes commonly reported in the US and EU. These consisted of ribotyes 027, 106, 014, 002, 017, 001, 078, 036, 087. Toxin B assay was performed as described in Methods. The slope of the dose response curve was determined for each toxin using 6 different concentrations of purified toxin B (16 pg/mL to 1250 pg/mL) with 6 replicates performed at each concentration. As shown in the Table S1, the slope of dose response curve varied from 3.6 to 14.6 indicating that all the toxins could be detected using MultiPath assays.

**Table S1: Slope of dose response curve.**

| Ribotype | Slope of Dose Response |
| --- | --- |
| 087 | 9.1 |
| 027 | 6.2 |
| 106 | 5.4 |
| 014 | 3.6 |
| 002 | 8.9 |
| 017 | 8.7 |
| 001 | 6.2 |
| 078 | 4.2 |
| 036 | 14.6 |

***Interference studies****.* The *C. difficile* toxin B MultiPath assay interference studies were carried out using twenty (20) potentially interfering substances that are commonly associated with diarrheal stool samples (listed in Table S2). Each interfering substance was evaluated at its "worst case" physiologically relevant concentrations. Using a pool stool sample (8% sample concentration), each substance was tested with and without a spike of 1.25ng/mL toxin B in replicates of 5. The results, shown in Table S2, indicate that none of the substances tested showed an inhibitory effect of the detection of C. *difficile* toxin B or caused a false positive result in the negative condition.

**Table S2: Assay interference testing**

|  |  | **Change in call** | |
| --- | --- | --- | --- |
| **Substance** | **Concentration** | **Negative** | **Positive** |
| Aluminum hydroxide w/magnesium hydroxide (Mylanta) | 4mg/mL | No | No |
| Barium Sulfate | 5mg/mL | No | No |
| Benzalkonium Chloride | 5mg/mL | No | No |
| Bismuth subsalicylate (Kaopectate) | 17.5mg/mL | No | No |
| Bismuth subsalicylate (Pepto-Bismol) | 17.5mg/mL | No | No |
| Blood 100% | 5% v/v | No | No |
| Calcium Carbonate | 5mg/mL | No | No |
| Ethanol 10% | 5% v/v | No | No |
| Hydrocortisone (Preparation H) | ~10mg/mL | No | No |
| Loperamide HCL (Imodium A-D) | 1mg/mL | No | No |
| Metronidazole | 12mg/mL | No | No |
| Mineral Oil- Light | 5% v/v | No | No |
| Mucin | 3.5mg/mL | No | No |
| Nonoxynol-9 | 70mg/mL | No | No |
| Nystatin | 5mg/mL | No | No |
| Phenylephrine HCL | 5mg/mL | No | No |
| Polyethylene Glycol | 80mg/mL | No | No |
| Simethicone | 1mg/mL | No | No |
| Sennosides (Ex-Lax) | 0.13mg/mL | No | No |
| Vancomycin HCL | 12.5mg/mL | No | No |

***Cross-reactivity/Analytical specificity studies****.* The *C. difficile* toxin B MultiPath assay cross reactivity studies were carried out using 23 commonly encountered off-target species (Table S3). Each strain was evaluated at ≥ 1e8 CFU/mL. Using a pool stool sample, each strain was tested with and without the presence of 1.25ng/mL toxin B in replicates of 5. The results, shown in Table 3, indicated that none of the species tested showed any inhibitory effect of the detection of *C.* *difficile* toxin B or caused a false positive result in the negative condition due to cross reactivity.

**Table S3: Cross-reactivity/Analytical specificity studies**

| **No**. | **Strain tested** | **Change in call** | |
| --- | --- | --- | --- |
|  |  | **Negative** | **Positive** |
| 1 | *Bacillus cereus* | No | No |
| 2 | *Bacillus subtilis* | No | No |
| 3 | *Campylobacter jejuni* | No | No |
| 4 | *Candida albicans* | No | No |
| 5 | *Enterobacter cloacae* | No | No |
| 6 | *Enterococcus faecalis* | No | No |
| 7 | *Escherichia coli* | No | No |
| 8 | *Escherichia coli O157:H7* | No | No |
| 9 | *Klebsiella pneumoniae* | No | No |
| 10 | *Listeria monocytogenes* | No | No |
| 11 | *Pseudomonas aeruginosa* | No | No |
| 12 | *Salmonella enterica* NR13555 | No | No |
| 13 | *Salmonella enterica* NR4296 | No | No |
| 14 | *Salmonella enterica* NR4314 | No | No |
| 15 | *Salmonella enterica* NR4291 | No | No |
| 16 | *Serratia marcescens* | No | No |
| 17 | *Shigella dysenteriae* | No | No |
| 18 | *Shigella flexneri* | No | No |
| 19 | *Shigella sonnei* | No | No |
| 20 | *Staphylococcus aureus* | No | No |
| 21 | *Staphylococcus aureus Cowen* | No | No |
| 22 | *Staphylococcus epidermis* | No | No |
| 23 | *Yersinia enterocolitica* | No | No |
